# An Aberrant Case of Neuromyelitis Optica Spectrum Disorder With a Review of Literature

**DOI:** 10.7759/cureus.59765

**Published:** 2024-05-06

**Authors:** Avinash Dhok, Chetana Ratnaparkhi, Santha Kumar, Smarth D Manhas, Ashwini Umredkar

**Affiliations:** 1 Department of Radiodiagnosis and Interventional Radiology, All India Institute of Medical Sciences (AIIMS) Nagpur, Nagpur, IND; 2 Department of Radiology, All India Institute of Medical Sciences (AIIMS) Nagpur, Nagpur, IND; 3 Department of Imaging Sciences and Interventional Neuroradiology, Sree Chitra Tirunal Institute for Medical Sciences & Technology (SCTIMST), Thiruvananthapuram, IND; 4 Department of Radiodiagnosis, All India Institute of Medical Sciences (AIIMS) Nagpur, Nagpur, IND

**Keywords:** anti-aquaporin 4 (aqp4) antibody, myelin-oligodendrocyte glycoprotein antibody-associated disease, conus medullaris lesions, acquired demyelinating disorder, optic neuritis, spinal cord inflammation, neuromyelitis optica spectrum disorder

## Abstract

Neuromyelitis optica spectrum disorder (NMOSD) is a rare, acquired demyelinating condition predominantly affecting middle-aged women and is characterized by spinal cord inflammation and optic neuritis. Anti-aquaporin 4 (AQP4) antibodies are typically seen in NMOSD. However, myelin oligodendrocyte glycoprotein antibody-associated disease (MOGAD) shares clinical and imaging similarities. In NMOSD, longitudinally extensive spinal cord lesions (LESCLs), optic neuritis predominantly affecting the posterior aspect of optic nerves, and optic radiations are seen on magnetic resonance imaging (MRI). The brain parenchymal lesions particularly involve the dorsal medulla (area postrema).

The report presents a case of a 26-year-old female with recurrent episodes of weakness, pain, and sensory symptoms in both upper and lower limbs who was initially treated for multiple sclerosis. Upon experiencing new symptoms of blurred vision and ataxia, an MRI of the spine and brain was performed, which showed short-segment cervical cord involvement and a lesion in the conus medullaris, raising the suspicion of NMOSD. Subsequent antibody testing confirmed the presence of anti-AQP4 antibodies. While the involvement of the conus medullaris is classically associated with MOGAD, unusual findings in the present case highlight the importance of comprehensive imaging evaluation and raising awareness among clinicians and radiologists regarding the imaging spectrum of NMOSD, thus facilitating timely diagnosis and tailored treatment strategies.

## Introduction

Neuromyelitis optica spectrum disorder (NMOSD) is a chronic inflammatory autoimmune condition of the central nervous system (CNS). The prevalence of NMOSD is approximately 0.3 to 4.4 per 100,000 population [1]. It classically presents with long-segment spinal cord inflammation, severe optic neuritis, and/or bouts of intractable vomiting and hiccoughs (area postrema syndrome). The hallmark of NMOSD is the presence of antibodies against aquaporin 4 (AQP4) in the foot processes of astrocytes. The International Panel for NMOSD Diagnosis (IPND) has stratified NMOSD based on the presence or absence of AQP4-IgG [2].

The present case is AQP4 positive NMOSD in a 26-year-old female with an unusual imaging appearance on magnetic resonance imaging (MRI) showing short-segment involvement of the conus medullaris. Conus medullaris involvement is more commonly seen in myelin oligodendrocyte glycoprotein antibody-associated Disease (MOGAD); however, the involvement of the conus medullaris in AQP4-positive NMOSD has been infrequently reported [3].

## Case presentation

A 26-year-old female presented with complaints of recurrent episodes of weakness, burning sensation, and fluctuating pain in both upper and lower extremities for two years, with partial remission. She also complained of recent onset blurring of vision and ataxia for five days. She was evaluated and treated for multiple sclerosis for previous episodes. The patient had not given a family history of such complaints.

On clinical examination, the deep tendon reflexes, pain, temperature, and crude and fine touch sensations were reduced in the left upper and lower limbs. There were no seizures, diplopia, nausea, vomiting, facial pain, drooling of saliva, or bowel and bladder dysfunction.

**Figure 1:**
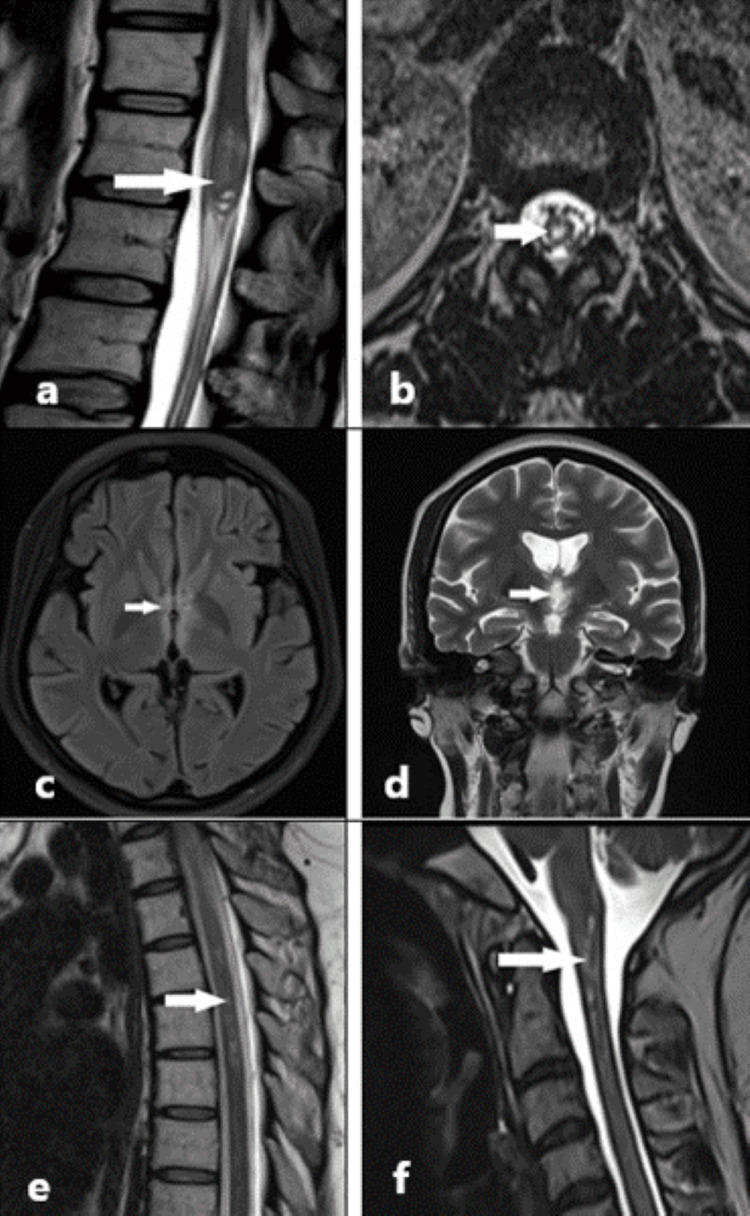
Magnetic resonance imaging of the spine and brain 1a: T2-weighted sagittal image of the spine at the level of the conus medullaris showing hyperintense areas in the conus medullaris (white arrow). 1b: T2-weighted axial image at the level of the conus medullaris showing hyperintensity in the conus medullaris (white arrow). 1c: Fluid-attenuated inversion recovery (FLAIR) sequence; axial image of the brain showing periventricular hyperintensity around the third ventricle (white arrow). 1d: T2-weighted coronal image of the brain showing periventricular hyperintensity around the third ventricle (white arrow). 1e: T2-weighted sagittal image of the spine showing long-segment hyperintensities in the dorsal spinal cord extending from the D2 to D6 vertebral levels (white arrow). 1f: T2-weighted sagittal image of the spine showing short-segment hyperintensities in the cervicomedullary junction and upper cervical spinal cord (white arrow).

The patient underwent an MRI of the brain and spine, which showed hyperintensities on T2-weighted and fluid-attenuated inversion recovery (FLAIR) sequences in the periventricular region of the third ventricle and left cerebral peduncle, bilateral optic radiations, and cervical spinal cord; long-segment involvement of the dorsal spinal cord from the superior end plate of the second dorsal to sixth dorsal vertebral bodies, and short-segment involvement of the conus medullaris with a few tiny cysts (Figure 1).

**Figure 2:**
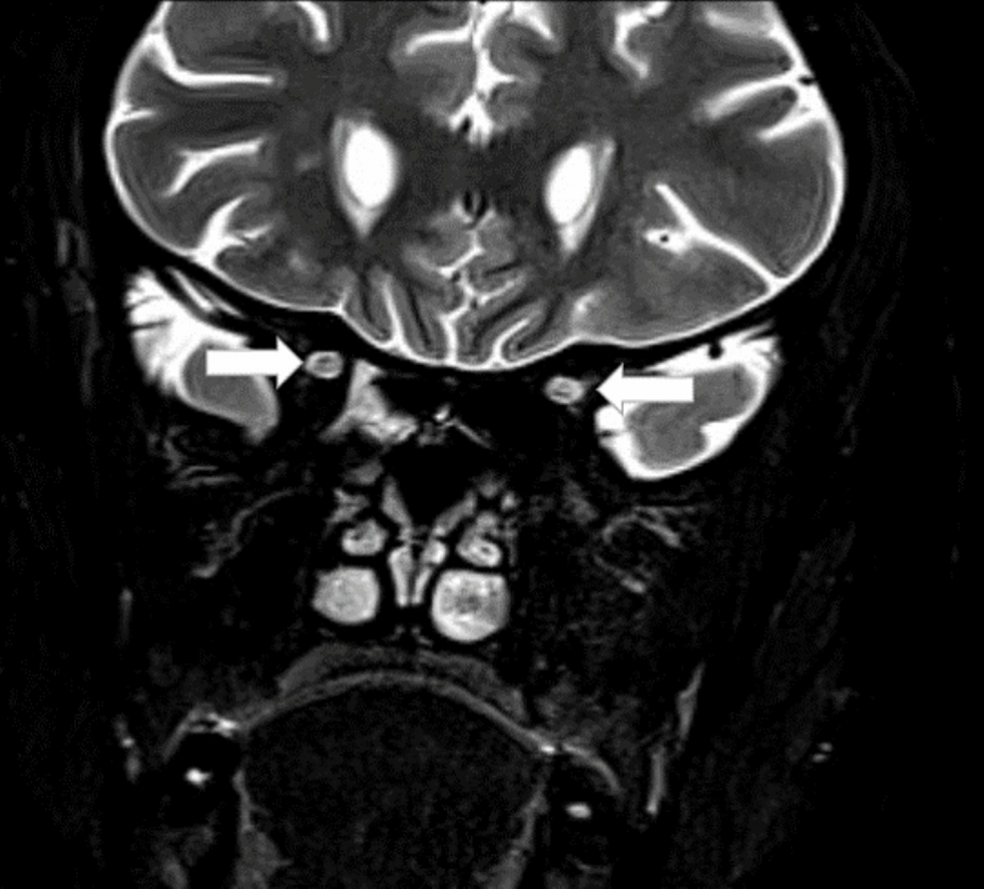
Coronal T2-weighted image of MRI orbit White arrows show hyperintensities in the posterior aspect of bilateral optic nerves.

There was also an involvement of the posterior aspect of both optic nerves (Figure 2).

**Figure 3:**
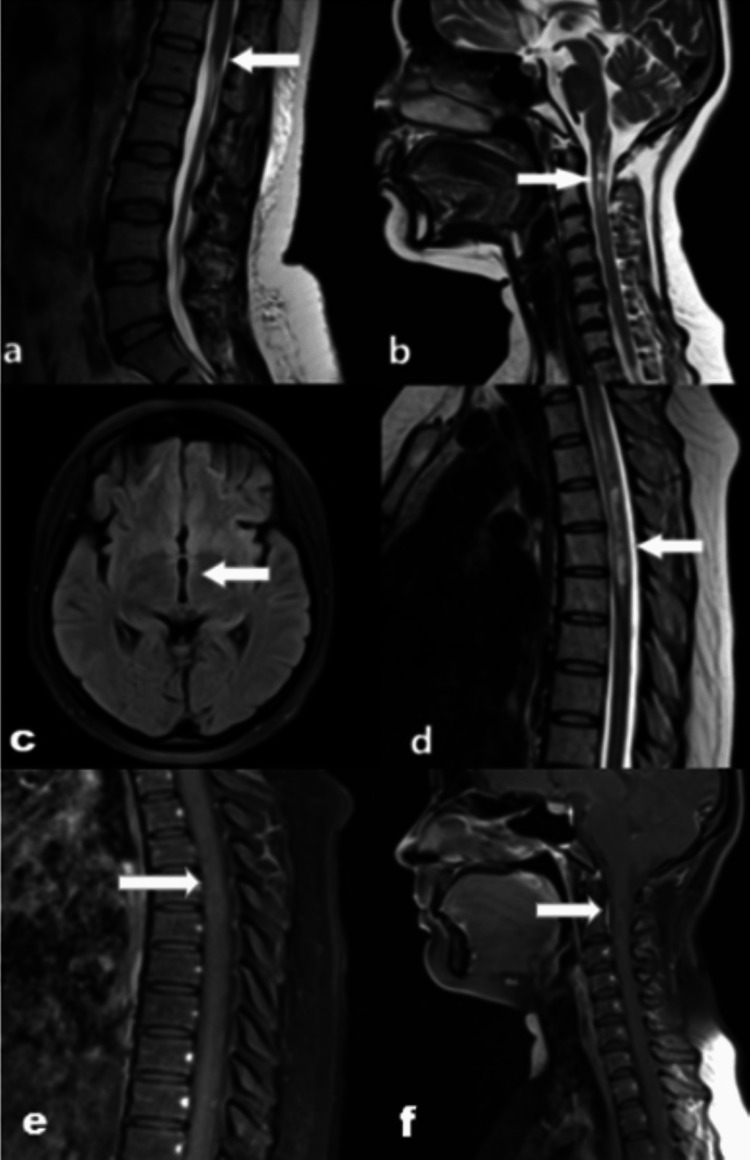
Previous magnetic resonance imaging of the spine and brain of the same patient done one year ago 3a: T2-weighted sagittal image of the spine at the level of the conus medullaris showing no abnormal signal intensity (white arrow). 3b: T2-weighted sagittal image showing short-segment hyperintensities in the cervicomedullary junction and upper cervical spinal cord (white arrow). 3c: T2-weighted axial image of the brain showing no abnormal signal intensities in the periventricular region of the third ventricle (white arrow). 3d: T2-weighted sagittal image of the spine showing long-segment hyperintensities in the dorsal spinal cord (white arrow). 3e: Post-contrast T1-weighted sagittal image of the spine showing no significant enhancement in dorsal spinal cord lesions (white arrow). 3f: Post-contrast T1-weighted sagittal image of the spine showing no significant enhancement in cervical spinal cord lesions (white arrow).

A previous MRI of the brain and spine done one year back revealed hyperintensities on T2-weighted and FLAIR sequences in the periventricular white matter of the frontal horn of the right lateral ventricle, short-segment involvement of the spinal cord from the cervicomedullary junction to the inferior end plate of the second cervical vertebral body and long-segment involvement of the dorsal spinal cord from the superior end plate of the second dorsal to sixth dorsal vertebral bodies (Figure 3).

No enhancement was seen in these areas on the post-contrast scan.

Compared to the previous MRI, the present MRI showed increased brain parenchymal involvement and involvement of bilateral optic nerves and conus medullaris. The current MRI findings were consistent with NMOSD; hence, aquaporin 4 antibody evaluation was suggested. Aquaporin 4 antibodies were reported positive, thus confirming the diagnosis of aquaporin 4 antibody-positive NMOSD.

Patient management and follow-up

The patient was administered methylprednisolone and later on shifted to oral prednisolone therapy. The patient showed improvement with treatment.

## Discussion

NMOSD is a rare autoimmune condition affecting predominantly middle-aged women and is characterized by longitudinally extensive myelitis and optic neuritis. Anti-AQP4 antibodies are noted in 70-80% of cases of NMOSD [4].

MRI features of NMOSD include bright, spotty, longitudinally extensive spinal cord lesions (LESCLs) involving mainly the central gray matter of the spinal cord, extending over more than three vertebral segments, optic neuritis involving predominantly the posterior aspect of optic nerves (intracanalicular and chiasmatic portions of the optic nerves), and optic radiations. Brain parenchymal lesions are also reported and often seen in the hypothalamus, periaqueductal grey matter, and dorsal medulla (area postrema) [5]. MOGAD is a rare, antibody-mediated inflammatory demyelinating disorder of the CNS presenting with clinical and imaging findings similar to NMOSD [3].

The present case had a history of recent-onset blurring of vision and ataxia along with previous episodes of weakness and burning pain in the left upper and lower limbs. MRI features of involvement of the periependymal area of the third ventricle, posterior portion of both optic nerves, and long-segment thoracic cord involvement favor NMOSD. Atypical imaging features in the present case were short-segment cervical cord and conus medullaris involvement. These MRI features are commonly seen in MOGAD [3].

A comparative study by Jain RS et al. in a patient showing demyelinating disorders observed that the involvement of the conus medullaris is characteristically seen in MOGAD as compared to anti-AQP4 antibody-positive NMSOD [6]. Another retrospective observational study by Kim HJ et al. and Salama S et al. found that involvement of the medulla, mainly the area postrema, cervicodorsal spinal cord, with an extension of the cervical lesion up to the brainstem was more common in anti-AQP4 antibody-positive NMOSD. On the contrary, the involvement of the upper brainstem (midbrain and pons), cortex, and conus medullaris was more common in the MOGAD group [5,7]. In NMOSD, involvement of the optic nerve is seen mainly in the posterior aspect with the involvement of the optic chiasm and optic radiation, while in MOGAD, it typically affects the anterior optic nerves, generally sparing the optic chiasm and tracts [8].

In the present case of anti-AQP4 antibody-positive NMSOD, the involvement of the conus medullaris was noted on MRI, which is an unusual finding. Additionally, short-segment involvement of the cervical cord (less than three vertebral segments) and conus medullaris was also an unusual finding contrary to NMOSD, which typically shows long-segment cord involvement.

However, optic nerve involvement is typical for NMOSD, i.e., the involvement of the posterior aspect of optic nerves (intracanalicular and chiasmatic portions of optic nerves) and optic radiations.

At times, NMOSD can show conus medullaris involvement, especially with other typical findings of NMOSD.

## Conclusions

NMOSD is a demyelinating disorder predominantly affecting middle-aged women with specific MRI features, viz., LESCLs, optic neuritis, and brain parenchymal lesions particularly involving the dorsal medulla (area postrema). The involvement of the conus medullaris is a specific imaging feature of MOGAD but can also be seen in patients with anti-AQP4 antibody NMSOD, as seen in the current case. Furthermore, short-segment cord involvement can sometimes be seen with NMOSD. Hence, radiologists should be aware of this unusual finding while evaluating patients with demyelinating disorders, as the diagnosis is based on complete imaging features along with laboratory findings.
